# Draft Genome Sequence of *Mycobacterium neoaurum* Strain DSM 44074^T^

**DOI:** 10.1128/genomeA.00699-14

**Published:** 2014-07-10

**Authors:** Michael Phelippeau, Catherine Robert, Olivier Croce, Didier Raoult, Michel Drancourt

**Affiliations:** Unité de Recherche sur les Maladies Infectieuses et Tropicales Émergentes, CNRS, UMR 7278, IRD 198, Faculté de Médecine, Aix-Marseille Université, Marseille, France

## Abstract

We report the draft genome sequence of *Mycobacterium neoaurum* strain DSM 44074^T^, a nontuberculosis species responsible for opportunistic infections in immunocompromised patients. The strain described here is composed of 5,536,033 bp, with a G+C content of 66.24%, and carries 5,274 protein-coding genes and 72 RNA genes.

## GENOME ANNOUNCEMENT

*Mycobacterium neoaurum* is an environmental, rapidly growing mycobacterium that is rarely reported as an opportunistic pathogen responsible for bloodstream infections, cutaneous infections, and pneumonia in immunocompromised patients (1, 2). To complete a genome sequence derived from an environmental Russian isolate (3) we sequenced the whole genome of *M. neoaurum* DSM 44074^T^, a strain confirmed as a valuable representative of the species (1).

Genomic DNA was isolated from *M. neoaurum* strain DSM 44074^T^ grown on MGIT broth medium (Becton Dickinson, Le Pont-de-Claix, France) at 37°C in an atmosphere enriched with 5% CO_2_. Genomic DNA was then sequenced using two high-throughput next-generation sequencing technologies: Roche 454 (4) and MiSeq Illumina (Illumina Inc., San Diego, CA). A 5-kb paired-end library was constructed, loaded on a PTP plate, and sequenced with the Roche-GS FLX Titanium sequencing kit XLR70, which produced 157,956 reads. Illumina sequencing was performed using two mate-pair Nextera libraries, sequenced on MiSeq in 2 × 250 bp. The DNA fragments ranged in size from 1 to 10 kb, and final sequencing produced a total of 145,157 reads. Reads from various sequencing technologies were first assembled separately. Reads from Roche 454 sequencing technologies were assembled into contigs and scaffolds using Newbler version 2.8 (Roche, 454 Life Sciences). Illumina reads were trimmed using Trimmomatic (5) and then assembled with Spades software (6, 7) while contigs generated from Roche 454 were added. Contigs obtained were combined by SSPACE (8) and Opera software (9) and then combined by GapFiller version 1.10 (10) to reduce the set. Some manual refinements using CLC Genomics version 7 software (CLC bio, Aarhus, Denmark) and some homemade Python scripts improved the genome. Finally, the draft genome of *M. neoaurum* consists of 10 scaffolds of 45 contigs containing 5,504,703 bp and has an estimated size of 5,536,033 bp, including gaps. The G+C content of this genome is 66.24%.

Noncoding genes and miscellaneous features were predicted using RNAmmer (11), ARAGORN (12), Rfam (13), PFAM (14), and Infernal (15). Coding DNA sequences (CDSs) were predicted using Prodigal (16), and functional annotation was achieved using BLAST+ (17) and HMMER3 (18) against the UniProtKB database (19). The genome was shown to encode at least 72 predicted RNAs, including 5 rRNAs, 49 tRNAs, 1 transfer-messenger RNA, and 17 miscellaneous RNAs. A total of 5,274 identified genes have a coding capacity of 5,112,765 bp (coding percentage: 92.35%), including 239 (4.53%) genes found encoding putative proteins and 822 (15.59%) assigned as encoding hypothetical proteins. Moreover, 5,220 genes matched at least one sequence in the Clusters of Orthologous Groups (COG) database (20, 21) with BLASTp default parameters.

This report illustrates the genomic variability within *M. neoaurum* (3), a feature on which to base further evaluations.

### Nucleotide sequence accession numbers.

The *M. neoaurum* DSM 44074^T^ strain genome sequence has been deposited at EMBL under the accession numbers LK021337 to LK021346. The whole-genome shotgun master numbers are CCDR010000001 to CCDR010000045.
